# Revisiting the Determination of the Degree of Deacetylation Using Potentiometric Titration: A New Equation for Modified Chitosan

**DOI:** 10.3390/molecules29132962

**Published:** 2024-06-21

**Authors:** Ons Amamou, Sarah Kefil, Jean-Philippe Denis, Taoufik Boubaker, Sébastien Cardinal

**Affiliations:** 1 Département de Biologie, Chimie et Géographie, Université du Québec à Rimouski, Rimouski, QC G5L 3A1, Canada; 2 Laboratoire de Chimie Hétérocyclique, Produits Naturels et Réactivité (LR11S39), Faculté des Sciences, Université de Monastir, Monastir 5000, Tunisia

**Keywords:** chitosan, degree of deacetylation (*DDA*), determination of *DDA* of modified chitosan, potentiometric titration, naphtalene–chitosan derivatives

## Abstract

Chitosan is a biopolymer that can be subjected to a variety of chemical modifications to generate new materials. The properties of modified chitosan are affected by its degree of deacetylation (*DDA*), which corresponds to the percentage of D-glucosamine monomers in its polymeric structure. Potentiometric titration is amongst the simplest, most readily available, and most cost-effective methods of determining the *DDA*. However, this method often suffers from a lack of precision, especially for modified chitosan resins. This is in large part because the equation used to calculate the *DDA* does not consider the molecular weight of the chemically modified monomeric units. In this paper, we introduce a new equation that is especially suited for modified chitosan bearing three different types of monomers. To test this equation, we prepared naphthalene–chitosan resins and subjected them to potentiometric titration. Our results show that our new equation, which is truer to the real structure of the polymeric chains, gives higher *DDA* values than those of the routinely used equations. These results show that the traditional equations underestimate the *DDA* of modified chitosan resins.

## 1. Introduction

Chitin is the second most abundant polysaccharide produced in nature, after cellulose [1]. Chitosan is a natural biopolymer that is the product of the partial deacetylation of chitin. Polymeric chains of chitosan are linear (monomers are chained together by a β 1-4 glycosidic linkage) and composed of two randomly distributed monomers: D-glucosamine and N-acetylglucosamine. The ratio of the two monomers can vary greatly between two chitosan samples, depending on their natural origin or manufacturer. The degree of deacetylation (*DDA*) is used to express the monomer ratio. The *DDA* is the percentage of D-glucosamine units found in a chitosan sample (of the total of monomer units).

Due to its remarkable properties, including its non-toxicity, biocompatibility, biodegradability, and antimicrobial properties, chitosan has become an important research topic [2,3,4,5]. It has applications in medicine, cosmetics, agriculture, and textiles [6,7,8,9].

There are a variety of reactive functional groups in chitosan; thus, a variety of chemical transformations can be performed on this polymer, notably imination, carboxylation, alkylation, acylation, sulfonation, and cross-linking [10]. These modifications aim to improve the physical and chemical properties of the polymer for specific applications [11,12,13]. However, the chemical, physical, and biological properties of modified chitosan depend on the percentage of D-glucosamine monomers remaining in its polymeric structure (i.e., its *DDA*). Thus, the accurate determination of the *DDA* of modified chitosan can be critical for some applications. Furthermore, a comparison between the *DDA* values before and after the chemical modification can also constitute a very valuable tool for evaluating the level of modification applied to a chitosan resin [14].

Various methods have been reported to determine the *DDA* values of unmodified and modified chitosan resins, notably infrared spectroscopy, potentiometric titration [15,16,17], UV–vis spectroscopy [18], elemental analysis [19], and ^1^H and ^13^C NMR spectroscopy [20]. ^1^H NMR is generally viewed as the most accurate method of determining the *DDA* of a chitosan sample [21,22]. However, this technique is not always available, as it requires costly infrastructure. Furthermore, modified chitosan resins are often hardly soluble in any commercial deuterated solvent and, thus, require the use of solid-state NMR, a significantly less accessible technique.

The potentiometric titration introduced by Broussignac [15] is simple, readily available, and cost-effective. Many research groups have employed this method to evaluate the *DDA* value of modified chitosan resins [23,24]. However, this method often suffers from a lack of precision, especially in the case of heavily modified chitosan resins [25]. This is partly because the equation used to calculate the experimental data obtained through titration (a volume of NaOH) into the *DDA* does not include the molecular weight of the modified monomeric units.

To circumvent this limitation, we report herein a new equation especially suited for the determination of the *DDA* of modified chitosan. To test the reliability of our equation, three series of new naphthalene–chitosan resins were prepared and submitted to potentiometric titration.

## 2. Results and Discussion

**Figure 1:**
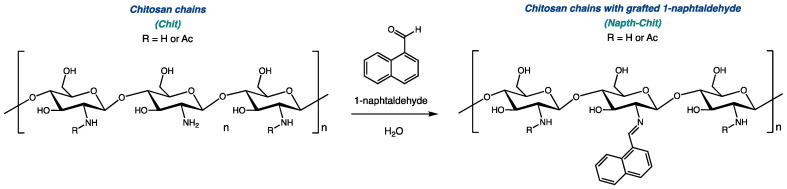
Derivatization of chitosan with 1-naphtaldehyde.

As we worked to develop new bioactive biosourced materials, we were interested in modifying chitosan with naphthalene groups. Previous research has shown that adding aromatic substituents to chitosan could lead to enhanced antimicrobial activity [26,27]. As illustrated in Figure 1, 1-naphtaldehyde, like most aldehydes, can be grafted to the polymeric chain of chitosan via the transformation of a Schiff base [28,29,30].

Three series of chitosan grafted with naphtalene (*Napth-Chit*) were prepared from three commercially available high-grade chitosan resins (ChitoClear^®^ HQG, Primex ehf, Siglufjörður, Iceland). To prepare *Napht-Chit* resins with various levels of modification, each of the three starting materials was divided into five parts, and each fraction was submitted to a different molar ratio of 1-naphtaldehyde. To establish the stoichiometry of those reactions, only the monomeric units with free NH_2_ groups were considered for chitosan, so the ratio measured was that of 1-naphtaldehyde to free amines (Napht:NH_2_ ratio). Five different ratios were explored, ranging from 1:4 to 4:1. The installation of the naphthalene moiety on all of the modified resins was assessed using FT-IR studies (see the Supplementary Materials for details). Table 1 shows the chitosan resins (starting materials and modified resins) involved in this study.

**Figure 2:**
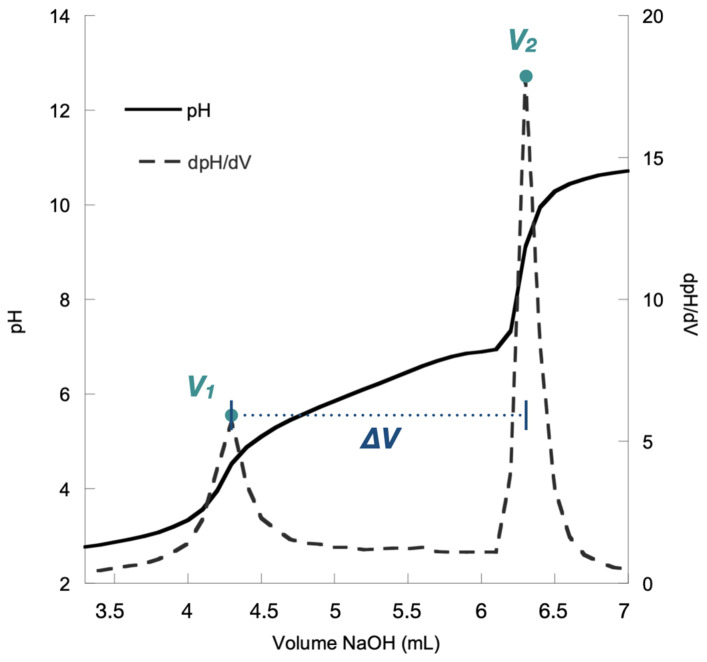
Titration curve for the chitosan resin *Napht-Chit-2a*.

Potentiometric titration was performed in triplicate on all of the chitosan resins presented in Table 1. Figure 2 shows an example of a titration curve for one of our resins.

As mentioned earlier, this method of characterization is simple and straightforward. In brief, the chitosan resin was first dissolved in an aqueous HCl solution to ensure that all of the amino groups (NH_2_) were protonated to their ammonium form (NH_3_^+^). Then, the resulting solution was titrated with aqueous NaOH. During titration, two inflection points were recorded after the addition of two volumes of aqueous NaOH (*V*_1_ and *V*_2_, Figure 2).

The first inflection point (associated with volume *V*_1_) corresponded to the volume necessary to neutralize the excess HCl used to dissolve the chitosan resin. The second inflection point (associated with volume *V*_2_) was observed when the ammonium functionalities (NH_3_^+^) on the resin were deprotonated to their amino form (NH_2_). Thus, the difference between *V*_2_ and *V*_1_ gave the quantity of NaOH necessary for the neutralization of the ammonium functions of the chitosan. This difference (*V*_2_ − *V*_1_), which we label as Δ*V* (Figure 2), was the experimental data used for the determination of the *DDA* of the chitosan resin.

The raw data obtained from titration (Δ*V*) could not be used to directly determine the value of the *DDA*, as it needed to be calculated from an equation involving a series of other variables. In the literature, two equations are routinely used to determine the *DDA* of modified chitosan resins from a titration curve.

The first equation was notably used by Beppu and Souza to determine the *DDA* of glutaraldehyde and epichlorohydrin cross-linked chitosan resins [23,24]. This equation is the simplest, as it considers the chitosan resin to be a homopolymer of D-glucosamine. It is labeled here as Equation (1):

(1) $$DDA\;(\%)=100\frac{\left(161.16\;g/mol\times ∆V\times [NaOH]\right)}{m}$$

for which [*NaOH*] is the concentration (mol/L) of the sodium hydroxide solution used for titration, Δ*V* (*V*_2_ − *V*_1_) is the volume of this NaOH solution (L) required to neutralize the ammonium functionalities, 161.16 (g/mol) is the molecular weight of the D-glucosamine monomer, and *m* is the mass (g) of the sample in the dry state before titration.

The second equation was introduced by Broussignac over 50 years ago [15]. In recent years, it has notably been used by Eldin [26], Tamer [27], and Misran [31] to determine the *DDA* of the Schiff base derivatives of aromatic and phenolic chitosan or acylated low-molecular-weight chitosan resins. It is labeled here as Equation (2):

(2) $$DDA\;(\%)=100\frac{\left(203.19\;g/mol\times ∆V\times [NaOH]\right)}{m+\left(42.03\;g/mol\times ∆V\times [NaOH]\right)}$$

for which [*NaOH*] is the concentration (mol/L) of the sodium hydroxide solution used for titration, Δ*V* (*V*_2_ − *V*_1_) is the volume (L) of this NaOH solution required to neutralize the ammonium functionalities, 203.19 (g/mol) is the molecular weight of the N-acetyl-glucosamine monomer, 42.03 (g/mol) is the difference between the molecular weight of the N-acetyl-glucosamine monomer and that of the D-glucosamine monomer, and *m* is the mass (g) of the sample in the dry state before titration.

Using Equations (1) and (2), we were able to calculate the *DDA* values for our chitosan resins from our titration results. The results are shown in Table 2. A significant difference in the *DDA* values was observed between the starting materials and their corresponding derivatized analogs. As expected, for each series, the increase in the Napht:NH_2_ ratio used during derivatization (from *Napht-Chit-Xa* to *Napht-Chit-Xe*) led to a decrease in the *DDA*. In other words, the use of a larger molar equivalent of 1-naphtaldehyde led to the installation of more naphthaldehyde moieties on the polymeric chains and, thus, a decrease in the number of free amines.

As shown in Table 2, both equations resulted in a significant difference in the *DDA* values for each chitosan resin. Overall, the difference (Δ*DDA* in Table 2) ranged between 4% and 5.1% for unmodified chitosan and between 5% and 6% for modified chitosan. This was not surprising, since these two equations treated the makeup of the polymeric chains differently. As mentioned above, Equation (1) considered that the chitosan chains were only composed of D-glucosamine, while Equation (2) considered the presence of the two monomers of natural chitosan: D-glucosamine and N-acetyl-glucosamine. In that sense, Equation (2) gave a more realistic evaluation of the *DDA* than that of Equation (1), especially for unmodified chitosan.

However, both of these equations are far from optimal for accurately determining the *DDA* of modified chitosan resins, as they do not consider that the chemical modification of the biopolymer brings at least one new type of monomer (with its distinct molecular weight) to the polymeric chain. In the case of the present study, the naphtalene Schiff base derivative of glucosamine (labeled as N-Napht-glucosamine in Table 3) that was introduced as the new monomer had a molecular weight that was almost twice that of D-glucosamine (Table 3). Neglecting this difference inevitably led to a loss of accuracy when determining the *DDA*.

To circumvent this limitation and to ensure that potentiometric titration remains a reliable technique for the determination of the *DDA* of modified chitosan resins, we decided to develop our own equation.

To introduce this new equation, we must first recall that the number of amino groups in the analyzed chitosan sample will be the same as the number of ammonium groups that is determined through the titration of that same chitosan sample. When expressed as molar quantities, this equality yields Equation (3):

(3) $$n_{NH3+}=n_{NH2}$$

for which *n_NH_*_3+_ is the number of moles of the ammonium group determined through titration, and *n_NH_*_2_ is the number of moles of the amino group present in the chitosan sample.

As discussed earlier, the difference between the two inflexion points observed during the titration of a chitosan sample (Δ*V*) gives the quantity of NaOH necessary for the neutralization of the ammonium functions of the chitosan. For *n_NH_*_3+_, this relation can be expressed as Equation (4):

(4) $$n_{NH3+}=∆V\times [NaOH]$$

for which *n_NH_*_3+_ is the number of moles of the ammonium group determined through titration, Δ*V* is the difference between the two inflexion points (L), and [*NaOH*] is the concentration of the sodium hydroxide solution used for titration (mol/L).

The other term of Equation (3), *n_NH_*_2_, can also be detailed, giving Equation (5):

(5) $$n_{NH2}=\frac{m_{chit}\times DDA}{M_{chit}}$$

for which *m_Chit_* is the mass of the chitosan sample used in the titration (g), *DDA* is the degree of deacetylation of the modified resin (%), and *M_chit_* is the molar mass of the chitosan resin (g/mol).

For a modified chitosan resin that has three types of monomers (D-glucosamine, *N*-acetyl-D-glucosamine, and the new type of monomer introduced by the chemical modification of chitosan), the value of *M_chit_* can be determined with Equation (6):

(6) $$M_{Chit}=\left(M_{gluc}\times DDA\right)+\left(M_{N-ac-gluc}\times DA\right)+\left(M_{mod-mono}\times ({DDA}_{SM}-DDA)\right)$$

for which *DDA* is the degree of deacetylation of the chitosan resin after the chemical modification, *M_glu_*_c_ is the molecular weight of the D-glucosamine monomer (161.16 g/mol), *M_N-ac-gluc_* is the molecular weight of the N-acetyl-glucosamine monomer (203.19 g/mol), *DA* is the degree of acetylation of the resin (%, *DA* = 1 − *DDA_SM_*), *DDA_SM_* is the degree of acetylation of the chitosan starting material used for modification (%), and *M_mod-mono_* is the molecular weight of the modified monomer (g/mol).

Applying Equations (5) and (6) gives Equation (7):

(7) $$n_{NH2}=\frac{m_{chit}\times DDA}{\left(M_{gluc}\times DDA\right)+\left(M_{N-ac-gluc}\times DA\right)+\left(M_{mod-mono}\times ({DDA}_{SM}-DDA)\right)}$$

From there, Equations (7) and (4) can be used to substitute the terms *n_NH_*_2_ and *n_NH_*_3+_ in Equation (3). This new equality gives Equation (8):

(8) $$∆V\times \left[NaOH\right]=\frac{m_{chit}\times DDA}{\left(M_{gluc}\times DDA\right)+\left(M_{N-ac-gluc}\times DA\right)+\left(M_{mod-mono}\times ({DDA}_{SM}-DDA)\right)}$$

Isolation of the *DDA* term from Equation (8) can then be performed in a few steps, as shown below with Equations (9)–(13).

(9) $$m_{chit}\times DDA=\left(∆V\times \left[NaOH\right]\times M_{gluc}\times DDA\right)+\left(∆V\times \left[NaOH\right]\times M_{N-ac-gluc}\times DA\right)\;+\left(∆V\times \left[NaOH\right]\times M_{mod-mono}\times {DDA}_{SM}\right)\;-\left(∆V\times \left[NaOH\right]\times M_{mod-mono}\times DDA\right)$$

(10) $$\left(m_{chit}\times DDA\right)-\left(∆V\times \left[NaOH\right]\times M_{gluc}\times DDA\right)+\left(∆V\times \left[NaOH\right]\times M_{mod-mono}\times DDA\right)\;=\left(∆V\times \left[NaOH\right]\times M_{N-ac-gluc}\times DA\right)+\left(∆V\times \left[NaOH\right]\times M_{mod-mono}\times {DDA}_{SM}\right)$$

(11) $$DDA\left[m_{chit}-\left(∆V\times \left[NaOH\right]\times M_{gluc}\right)+\left(∆V\times \left[NaOH\right]\times M_{mod-mono}\right)\right]\;=\left(∆V\times \left[NaOH\right]\times M_{N-ac-gluc}\times DA\right)\;+\left(∆V\times \left[NaOH\right]\times M_{mod-mono}\times {DDA}_{SM}\right)$$

(12) $$DDA\left[m_{chit}+\left(∆V\times \left[NaOH\right]\right)\left(M_{mod-mono}-M_{gluc}\right)\right]\;=\left(∆V\times \left[NaOH\right]\right)\left(\left(M_{N-ac-gluc}\times DA\right)+\left(M_{mod-mono}\times {DDA}_{SM}\right)\right)$$

(13) $$DDA=\frac{\left(∆V\times \left[NaOH\right]\right)\left(\left(M_{N-ac-gluc}\times DA\right)+\left(M_{mod-mono}\times {DDA}_{SM}\right)\right)}{\begin{array}{c} \left[m_{chit}+\left(∆V\times \left[NaOH\right]\right)\left(M_{mod-mono}-M_{gluc}\right)\right] \end{array}}$$

To simplify this expression, we can introduce the term *ΔM*, which is defined by Equation (14):

(14) $$∆M=M_{mod-mono}-M_{gluc}$$

for which Δ*M* (g/mol) is the difference between the molar mass of the modified monomer and the D-glucosamine monomer, *M_gluc_* is the molecular weight of the glucosamine monomer (161.16 g/mol), and *M_mod-mono_* is the molecular weight of the modified monomer (g/mol).

Introducing Equation (14) into Equation (13) and replacing *M_N-ac-gluc_* with its actual value (203.19 g/mol) leads to Equation (15).

(15) $$DDA=\frac{\left(∆V\times \left[NaOH\right]\right)\left(\left(203.19\;g/mol\times DA\right)+\left(M_{mod-mono}\times {DDA}_{SM}\right)\right)}{m_{chit}+\left(∆V\times \left[NaOH\right]\times ∆M\right)}$$

From there, the final expression of the degree of deacetylation of the chitosan resin after the chemical modification (*DDA*, in %) can be expressed with Equation (16):

(16) $$DDA\;(\%)=100\frac{\left(∆V\times \left[NaOH\right]\right)\left(\left(203.19\;g/mol\times DA\right)+\left(M_{mod-mono}\times {DDA}_{SM}\right)\right)}{m_{chit}+\left(∆V\times \left[NaOH\right]\times ∆M\right)}$$

for which Δ*V* is the difference between the two inflection points (L), [*NaOH*] is the concentration of the sodium hydroxide solution used for titration (mol/L), 203.19 g/mol is the molecular weight of the N-acetylglucosamine monomer, *DA* is the degree of acetylation of the resin (%, *DA* = 1 − *DDA_SM_*), *M_mod-mono_* is the molecular weight of the modified monomer (g/mol), *DDA_SM_* is the degree of acetylation of the chitosan starting material used for modification, *m_Chit_* is the mass of the chitosan sample used in the titration (g), and *ΔM* is the difference between the molar mass of the modified monomer and the D-glucosamine monomer (g/mol).

Equation (16) offers a new alternative for determining the *DDA* of a modified chitosan resin with potentiometric titration. The presence of the terms *M_mod-mono_* and Δ*V* in this equation clearly shows that this new equation accounts for the presence of a third type of monomer in addition to D-glucosamine and N-acetylglucosamine.

Equation (16) can be universally used to determine the *DDA* of any modified chitosan resin with three different types of monomers, as long as the molecular weight of the monomer introduced by chemical modification is known.

In the case of our *Napht-Chit* resins, we know that the molecular weight of the *N-Napht-glucosamine is* 299.33 g/mol and that the variable Δ*M* is fixed at 138.17 g/mol (299.33 g/mol–161.16 g/mol). Thus, for this series of modified chitosan, the general Equation (16) can be refined to Equation (17):

(17) $$DDA\;(\%)=100\frac{\left(∆V\times \left[NaOH\right]\right)\left(\left(203.19\;g/mol\times DA\right)+\left(299.33\;g/mol\times {DDA}_{SM}\right)\right)}{\begin{array}{c} m_{chit}+\left(138.17\;g/mol\times ∆V\times \left[NaOH\right]\right) \end{array}}$$

for which Δ*V* is the difference between the two inflexion points (L), [*NaOH*] is the concentration of the sodium hydroxide solution used for titration (mol/L), *DDA_SM_* is the degree of acetylation (%) of the chitosan starting material (*Chit-1*, *Chit-2*, or *Chit-3*) used for modification, as determined with Equation (2) (see Table 2), *DA* is the degree of acetylation of the resin (%, *DA* = 1 – *DDA_SM_*), 203.19 g/mol is the molecular weight of the N-acetylglucosamine monomer, 299.33 g/mol is the molecular weight of the modified monomer (*N-Napht-glucosamine*), *m_Chit_* is the mass of the chitosan sample used in the titration (g), and 138.17 g/mol is the difference between the molar mass of the modified monomer and the D-glucosamine monomer.

**Figure 3:**
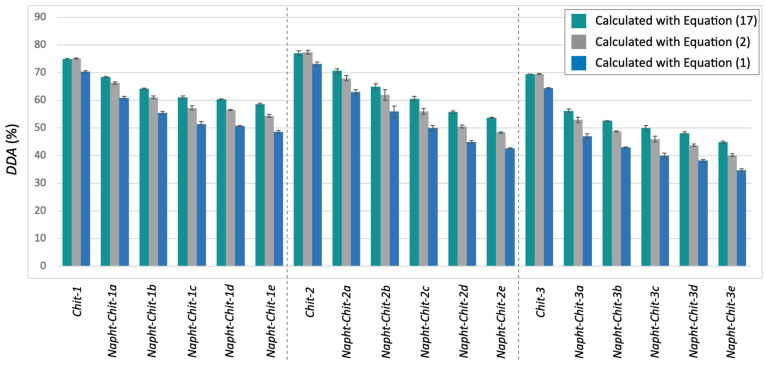
*DDA* values for all of the chitosan involved in this study as determined through potentiometric titration after calculation with Equation (17) (teal), Equation (2) (gray), and Equation (1) (blue).

Equation (17) was used to determine the *DDA* values for the chitosan resins involved in this study. The results are presented in Table 3. As a complement to Table 3, Figure 3 compares the *DDA* values obtained with Equation (17) with the other values previously calculated with Equations (1) and (2). It is noteworthy to mention that Equation (17) can also be used to determine the DDA of the starting material. No significant differences were observed between Equations (17) and (2) in those cases (see the Supplementary Materials and Figure 3).

Figure 3 illustrates that, as expected, the newly proposed Equation (17) gave higher *DDA* values for each modified chitosan resin. As detailed in Table 3, the difference between the mean *DDA* values calculated with Equations (2) and (17) ranged from 2.1 to 5.4%. This clearly indicated that even when using Equation (2) (the most accurate equation routinely used in the literature), the values of *DDA* obtained for modified chitosan from potentiometric titration tended to be underestimated.

In a related issue, this type of misinterpretation can lead to an overestimation of the degree of modification of the polymer if the *DDA* values calculated with Equation (2) before and after the modification are compared. As an example, consider *Napht-Chit-3c.* In this case, the *DDA* value of the starting material (*Chit-3*) is 69.5 ± 0.2% when calculated with Equation (2). The *DDA* value for *Napht-Chit-3c* is evaluated at 46 ± 1% or at 50 ± 1% according to Equation (2) or Equation (17), respectively. Thus, depending on the equation used, the percentage of naphthalene-grafted monomers that compose the structure of *Napht-Chit-3c* can be evaluated at 24 ± 1% [(69.5± 0.2) – (46 ± 1)] or 20 ± 1% [(69.5 ± 0.2) – (50 ± 1)], depending on which *DDA* value is used for the modified polymer.

Although this difference may not be crucial for many studies involving modified chitosan resins, some applications may require the most accurate method available. Furthermore, considering the nature of Equation (16), larger differences in the determination of the *DDA* will occur when (1) the molecular weight of the modified monomer increases and (2) the level of modification of the biopolymer (the amount of the D-glucosamine monomer that has been modified) increases. As illustrated in Figure 3, the latter trend was observed when applying Equation (17) to our *Napht-Chit* resins.

Improving the accuracy of potentiometric titration for the determination of the *DDA* of modified chitosan represents an important improvement, as this characteristic is often regarded as the weak point of the method. The present study shows that this limitation can be partly circumvented by using an equation that is more in line with the real molecular composition of the modified biopolymer.

Finally, our results show that the uncertainties in the DDA values calculated with Equation (17) are of the same order of magnitude as those obtained from the traditional equations. In all but one case, the uncertainty in the mean DDA value of the triplicate data calculated with Equation (17) was equal to or lower than (see Table 2 and Table 4) that calculated with either Equations (1) or (2). This is somewhat surprising considering that, because of the nature of Equation (17), the uncertainties in the values obtained with this equation must also consider the uncertainties in the variables *DDA_SM_* and *DA* (see the Supplementary Materials for a detailed example). Overall, this shows that the increase in accuracy of our new equation does not come at the expense of a decrease in reproducibility.

## 3. Materials and Methods

### 3.1. Materials

The three chitosan starting materials were purchased from Primex (Los Angeles, CA, USA): ChitoClear^®^ HQG 10 (Code product: 43000), ChitoClear^®^ HQG 400 (Code product: 43020), and ChitoClear^®^ HQG 1600 (Code product: 43040). All chemical reagents, including hydrochloric acid, naphtaldehyde, ethanol, acetone, sodium chloride, and sodium hydroxide, were purchased from MilliporeSigma (St. Louis, MA, USA) and used as purchased.

### 3.2. Preparation of Napth-Chit Resins

1-Naphtaldehyde-grafted chitosan resins with different levels of modification were prepared from ChitoClear^®^ HQG 10, ChitoClear^®^ HQG 400, and ChitoClear^®^ HQG 1600 using a procedure adapted from Gavalyan [32]. The three starting materials were divided into 5 fractions each and reacted with different concentrations of 1-naphtaldehyde, corresponding to various naphtaldehyde–free amine ratios (Napht:NH_2_). Ratios of 1:4, 1:2, 1:1, 2:1, and 4:1 were tested for each starting material (see the Supplementary Materials). Each combination (starting material/Napht:NH_2_ ratio) was performed in triplicate.

For each reaction, 1 g of lyophilized chitosan was added to 5 mL of distilled water in a conical centrifuge tube. Then, 4 mL of ethanol was added to each tube to solubilize the 1-naphtaldehyde. Next, a certain volume (calculated to reach the desired Napht:NH_2_ ratio and rounded to the closest 0.1 mL) of 1-naphtaldehyde was added, and the volume was topped up to 20 mL with water before the tube cap was tightly screwed. The reaction was stirred for 48 h at room temperature by using a benchtop laboratory shaker (SHAKER SK-71 Lab. Companion, at 230 rpm). The solution was then centrifuged at 3400× *g* for 6 min. The settled modified chitosan was rinsed 9 times with a volume of 40 mL of [sodium chloride 30 g/L (3×), distilled water (2×), acetone (1×), ethanol (1×), and distilled water (×2)] (with a 5 min agitation followed by centrifugation at 3400× *g* for 6 min). After the ninth rinse, the supernatant water was removed, and the remaining solid was lyophilized.

### 3.3. Potentiometric Titration of Chitosan Resins

All chitosan resins were lyophilized prior to titration. For each analysis, a solution of 50 mg of chitosan resin in 20 mL of diluted HCl (0.03 mol/L) was prepared and stirred for 30 min to allow sufficient time to charge all free amines. The resulting solution was then titrated with NaOH solution (0.1 mol/L). First, 3 mL of this solution was rapidly added to the sample. Then, the basic solution was added using the incremental addition of 100 μL every 5 min (total titration time = 3 h and 45 min). The volume of added NaOH and the pH values of the solution were recorded with an automatic titrator (848 Titrino plus, Metrohm, Herisau, Switzerland). Prior to each titration, calibration of the pH electrode was performed with buffer solutions of pH 4.00 and 7.00.

## 4. Conclusions

Potentiometric titration is one of the simplest methods to implement to determine the *DDA* of a chitosan sample. Although it offers great advantages in terms of accessibility and simplicity, this technique is generally considered to be less accurate than spectroscopic methods. This limitation can be particularly problematic for modified chitosan resins because the equations for converting the data obtained through titration (volumes) into *DDA* (%) found in the literature do not account for the changes induced in the molecular weight by the chemical modification of the natural biopolymer.

To circumvent this limitation, we introduced a new general equation to treat the data obtained from potentiometric titration and calculate the *DDA* of modified chitosan resins (with three types of monomers). To test this equation, we prepared three series of chitosan resins grafted with naphthalene starting from three different chitosan starting materials (different polymeric chain lengths). We demonstrated that our new equation, which is truer to the real structure of the polymeric chains than the routinely used equations, gives higher *DDA* values for all of the tested resins.

These results show that the data obtained from the potentiometric titration of modified chitosan resin, when calculated with the traditional equation, led to an underestimation of the *DDA*. This type of misinterpretation can also lead to an overestimation of the level of modification applied to the polymer if the chemical reaction applied to the chitosan resin involves the transformation of the amino group of D-glucosamine monomers. We are confident that our newly proposed equation will be adopted by the community of researchers devoted to the preparation of new chitosan-based materials, as it improves the accuracy of potentiometric titration—a very easy, accessible, and convenient technique for the determination of *DDA*, which is a key parameter of biopolymers.

## Figures and Tables

**Table 1:** Chitosan resins involved in this study: the identities of the three starting materials and the various naphtaldehyde-free amine ratios (Napht:NH_2_) explored for their modification.

Starting Material ^I^	Napht:NH_2_	Product Name
ChitoClear^®^ HQG 10	0 ^II^	*Chit-1*
	1:4	*Napht-Chit-1a*
	1:2	*Napht-Chit-1b*
	1:1	*Napht-Chit-1c*
	2:1	*Napht-Chit-1d*
	4:1	*Napht-Chit-1e*
ChitoClear^®^ HQG 400	0 ^II^	*Chit-2*
	1:4	*Napht-Chit-2a*
	1:2	*Napht-Chit-2b*
	1:1	*Napht-Chit-2c*
	2:1	*Napht-Chit-2d*
	4:1	*Napht-Chit-2e*
ChitoClear^®^ HQG 1600	0 ^II^	*Chit-3*
	1:4	*Napht-Chit-3a*
	1:2	*Napht-Chit-3b*
	1:1	*Napht-Chit-3c*
	2:1	*Napht-Chit-3d*
	4:1	*Napht-Chit-3e*

^I^ See the Supplementary Materials for details on the starting materials (MW and viscosity). ^II^ Unmodified chitosan.

**Table 2:** *DDA* values (%) of chitosan resins involved in this study calculated with classic equations from the potentiometric titration results.

Chitosan Resin	*DDA* Values Determined through Potentiometric Titration (%) ^I^		Δ*DDA* (%) ^III^
	Calculated with Equation (1)	Calculated with Equation (2)	
*Chit-1*	70.5 ± 0.2	75.1 ± 0.2	4.6 ± 0.4
*Napht-Chit-1a*	61.0 ± 0.5	66.3 ± 0.5	5 ± 1
*Napht-Chit-1b*	55.5 ± 0.5	61.1 ± 0.5	6 ± 1
*Napht-Chit-1c*	51.5 ± 0.8	57.3 ± 0.8	6 ± 2
*Napht-Chit-1d*	50.8 ± 0.2	56.5 ± 0.2	5.7 ± 0.4
*Napht-Chit-1e*	48.6 ± 0.5	54.4 ± 0.5	6 ± 1
*Chit-2*	73.2 ± 0.7	77.5 ± 0.7	4 ± 1
*Napht-Chit-2a*	63 ± 1	68 ± 1	5 ± 2
*Napht-Chit-2b*	56 ± 2	62 ± 2	6 ± 4
*Napht-Chit-2c*	50 ± 1	56 ± 1	6 ± 2
*Napht-Chit-2d*	44.9 ± 0.5 ^II^	50.6 ± 0.5 ^II^	6 ± 1
*Napht-Chit-2e*	42.6 ± 0.2 ^II^	48.4 ± 0.2 ^II^	5.8 ± 0.4
*Chit-3*	64.4± 0.2	69.5 ± 0.2	5.1 ± 0.4
*Napht-Chit-3a*	47 ± 1	53 ± 1	6 ± 2
*Napht-Chit-3b*	43.0 ± 0.2	48.8 ± 0.2	5.8 ± 0.4
*Napht-Chit-3c*	40 ± 1	46 ± 1	6 ± 2
*Napht-Chit-3d*	38.2 ± 0.5	43.8 ± 0.5	6 ± 1
*Napht-Chit-3e*	34.8 ± 0.5	40.2 ± 0.5	5 ± 1

^I^ See Supplementary Materials for the complete titration results. The means and standard deviations were calculated from triplicate data unless otherwise indicated. ^II^ Means and standard deviations of duplicate datasets. ^III^ *DDA*_calculated with Equation (2)_ − *DDA*_calculated with Equation (1)._

**Table 3:** Structure and molecular weight (g/mol) of the three monomers that composed the structure of the *Napht-Chit* chitosan resins prepared for this study.

Monomer	Structure	Molecular Weight (g/mol)
D-glucosamine	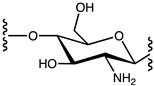	161.16
N-acetyl-glucosamine	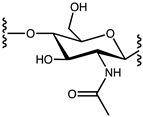	203.19
Modified monomer (N-Napht-glucosamine)	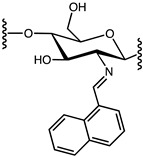	299.33

**Table 4:** *DDA* values (%) of the modified chitosan resins involved in this study calculated with the newly proposed Equation (17).

Chitosan Resin	*DDA* Values Calculated with the New Equation (17) (%) ^I^	Δ*DDA* (%) Equations (17)–(2)
*Napht-Chit-1a*	68.4 ± 0.3	2.1 ± 0.8
*Napht-Chit-1b*	64.2 ± 0.3	3.1 ± 0.8
*Napht-Chit-1c*	61.1 ± 0.6	4 ± 1
*Napht-Chit-1d*	60.4 ± 0.2	3.9 ± 0.4
*Napht-Chit-1e*	58.6 ± 0.4	4.2 ± 0.9
*Napht-Chit-2a*	70.7 ± 0.8	3 ± 2
*Napht-Chit-2b*	65 ± 1	3 ± 3
*Napht-Chit-2c*	60.6 ± 0.8	5 ± 2
*Napht-Chit-2d*	55.8 ± 0.4 ^II^	5.2 ± 0.9
*Napht-Chit-2e*	53.8 ± 0.2 ^II^	5.4 ± 0.4
*Napht-Chit-3a*	56.2 ± 0.7	3 ± 2
*Napht-Chit-3b*	52.6 ± 0.1	3.8 ± 0.3
*Napht-Chit-3c*	50 ± 1	4 ± 2
*Napht-Chit-3d*	48.2 ± 0.4	4.4 ± 0.9
*Napht-Chit-3e*	44.9 ± 0.4	4.7 ± 0.9

^I^ See the Supplementary Materials for the complete titration results. Means and standard deviations of triplicate datasets, unless otherwise indicated. ^II^ Means and standard deviations of duplicate datasets.

## Data Availability

The data presented in this study are available in the Supplementary Materials.

## Supplementary Materials

The following supporting information can be downloaded at https://www.mdpi.com/article/10.3390/molecules29132962/s1. 1. Information about the starting materials. 2. Preparation of *Napht-Chit resins*. 3. FTIR spectra of the chitosan resins involved in this study. 4. Potentiometric titration of the chitosan resins: (a) general principle; (b) titration results: complete raw values. 5. Determination of the *DDA* through titration: (a) using Equation (1): example; (b) using Equation (2): example; (c) calculating the Δ*DDA* [Equations (2) and (1)]: example; (d) using Equation (17): example; (e) calculating Δ*DDA* [Equations (17) and (2)]: example; (e) complete results. Supplementary Materials include: Table S1. Information on length of polymer chains [expressed by viscosity and molecular weight (MW)] and on the degree of deacetylation (DDASM, %) for the three chitosan starting materials used in this study; Table S2. Quantity of 1-naphtaldehyde used for the derivatization of 1g of starting material; Figure S1. FTIR spectra of Chit-1 and derivatives; Figure S2. FTIR spectra of Chit-2 and derivatives; Figure S3. FTIR spectra of Chit-3 and derivatives; Figure S4. Potentiometric determination of DDA in starting material ChitoClear® HQG 400; Table S3 Titration results for all the chitosan resins involved in this study; Table S4 Determination of DDA values for all the chitosan resins involved in this study as determined by potentiometric titration after calculation with Equation (1), Equation (2) and Equation (17): complete detailed results.
