# Supplementary material for: Revisiting the Determination of the Degree of Deacetylation Using Potentiometric Titration: A New Equation for Modified Chitosan
Source: Molecules. 2024 Jun 21;29(13):2962. doi: 10.3390/molecules29132962 (PMC11242947; doi:10.3390/molecules29132962)
Supplement: Supplementary file 1 [file molecules-29-02962-s001.zip › molecules-3036601-supplementary.pdf]

Supplementary materials

Revisiting the determination of the degree of deacetylation of modified chitosan resins using potentiometric titration

Ons Amamou [1,2], Sarah Kefil[1], Jean-Philippe Denis [1], Taoufik Boubaker [2] and Sébastien Cardinal [1,*]

[1]. Département de Biologie, Chimie et Géographie, Université du Québec à Rimouski, Rimouski, QC G5L 3A1, Canada
[2]. Laboratoire de Chimie Hétérocyclique, Produits Naturels et Réactivité (LR11S39), Faculté des Sciences, Université de Monastir, Monastir, Tunisia
*Correspondence: sebastien_cardinal@uqar.ca

Content

1. Information about the starting materials……………………………………………..…………………2

2. Preparation of *Napht-Chit resins*……………………………………………………………………...2

3. FTIR spectra of the chitosan resins involved in this study………………………………………………..3

4. Potentiometric titration of the chitosan resins……………………………..………………………... .5

a) General principle……………………………………………………………………………5

b) Titration results : complete raw values…………………..……………………………………....6

5. Determination of DDA from titration………...………………………..………………………... .8

a) Using Equation (1): example……………………………………………………………..…8

b) Using Equation (2): example……………………………………………………………..…9

c) Calculating ΔDDA [Equation (2)-Equation (1)] : example…………….……………………………10

d) Using Equation (17): example……………………………………………...…………….……10

e) Calculating ΔDDA [Equation (17)-Equation (2)] : example…………………………...………12

f) Complete results……………...…………………………………………………………………13

1. Information about the starting materials

Table S1. Information on length of polymer chains [expressed by viscosity and molecular weight (MW)] and on the degree of deacetylation (DDA_{SM}, %) for the three chitosan starting materials used in this study.

Commercial name	Viscosity (cps)[a]	Calculated MW (KDa)[I]	DDA_{SM} (%)[II]
ChitoClear® HQG 10 (Product code 43000)	<20	110-150	75.1 ± 0.2
ChitoClear® HQG 400 (Product code 43020)	200-600	250-300	77.5 ± 0.7
ChitoClear® HQG 1600 (Product code 43040)	1200-2000	340-360	69.5 ± 0.2

I. Data provided by the manufacturer.
II. Determined by potentiometric titration (means and standard deviations of triplicate). See Sections 4 and 5 of this supplementary materials for detailed titration results and determination of DDA.

2. Preparation of Napth-Chit resins

For each reaction, 1 g of lyophilized chitosan was added to 5 mL of distilled water in a conical centrifuge tube. Then, 4 mL of ethanol was added to each tube. Next, a certain volume (calculated to reach the desired Napht:NH_2 ratio and rounded to the closest 0.1 mL, see Table S2) of 1-naphtaldehyde was added, and the volume was topped up to 20 mL with water before the tube cap was tightly screwed. The reaction was stirred for 48 hours at room temperature by using a benchtop laboratory shaker (SHAKER SK-71 Lab. Companion, at 230 rpm). The solution was then centrifuged at 3400 g for 6 minutes. The settled modified chitosan was rinsed 9 times with a volume of 40 mL of [sodium chloride 30 g/L (3x), distilled water (2x), acetone (1x), ethanol (1x), and distilled water (x2)] (with a 5 min agitation followed by centrifugation at 3400 g for 6 minutes). After the ninth rinse, the supernatant water was removed, and the remaining solid was lyophilized.

Table S2. Quantity of 1-naphtaldehyde used for the derivatization of 1g of starting material.

Chitosan resin prepared	Starting material	Napht:NH_2 ratio	Volume of 1-naphatldehyde used for the reaction (mL)
Napht-Chit-1a	*Chit-1*	1:4	0.2
Napht-Chit-1b		1:2	0.4
Napht-Chit-1c		1:1	0.8
Napht-Chit-1d		2:1	1.6
Napht-Chit-1e		4:1	3.2
Napht-Chit-2a	*Chit-2*	1:4	0.2
Napht-Chit-2b		1:2	0.4
Napht-Chit-2c		1:1	0.8
Napht-Chit-2d		2:1	1.6
Napht-Chit-2e		4:1	3.2
Napht-Chit-3a	Chit-3	1:4	0.5
Napht-Chit-3b		1:2	1.0
Napht-Chit-3c		1:1	1.9
Napht-Chit-3d		2:1	3.8
Napht-Chit-3e		4:1	7.7

3. FTIR spectra of chitosan resins

FTIR spectra of the three series of chitosan resins (the starting materials and modified derivatives) are shown in Figures S1–S3. For the three series, four bands were used to confirm the formation of the imine functionalities between 1-naphtaldehyde and chitosan. Those four bands were absent from the IR spectrum of the starting materials. Furthermore, for each series of chitosan resins, those bands become more defined and intense as the level of functionalization increases from *Napth-Chit-Xa* to *Napht-Chit-Xe.* The first two bands around 770 and 800 cm^{-1} are attributed to the aromatic out-of-plane C-H bending. The third band around 1500 cm^{-1} is associated with C=C ring stretch. The fourth band around 1675 cm^{-1} is attributed to the C=N bond of the imine functionality. It is a bit more challenging to observe because unmodified chitosan shows a strong peak around 1645 cm^{-1} associated with the residual N-acetyl groups (C=O stretching of amide I).

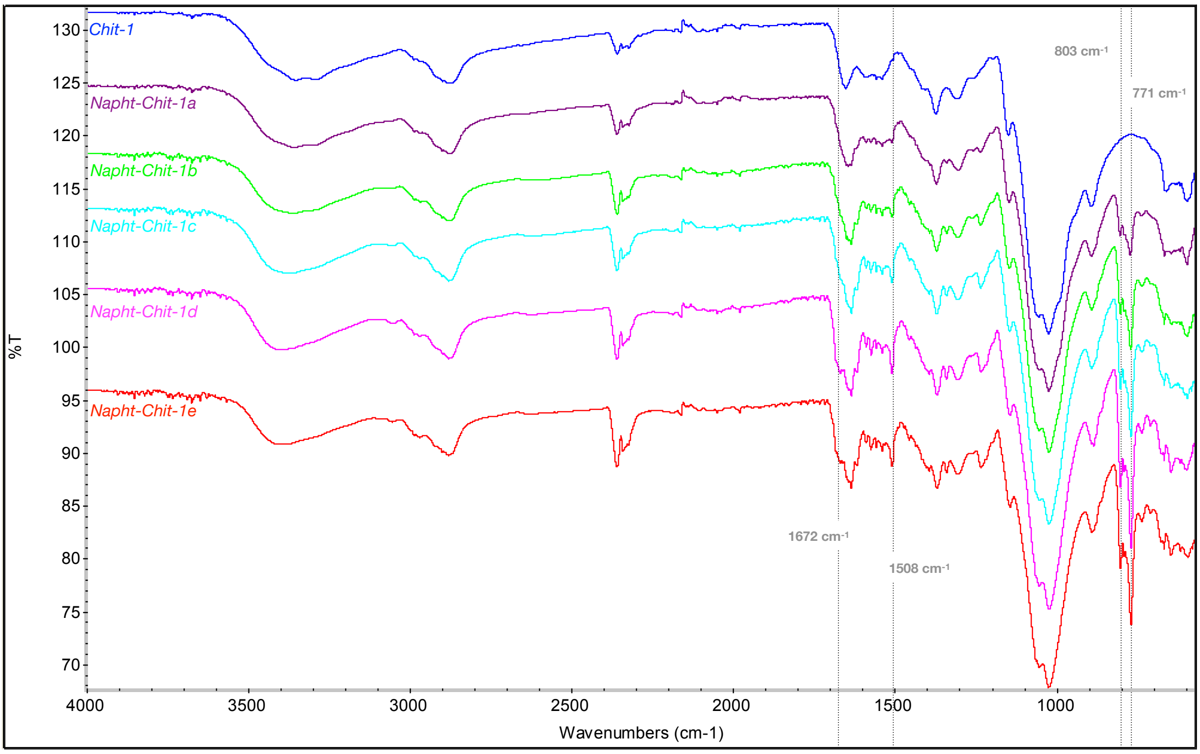

Figure S1. FTIR spectra of *Chit-1* and derivatives. From top to bottom: *Chit-1* (blue trace), *Napht-Chit-1a* (plum trace), *Napht-Chit-1b* (green trace), *Napht-Chit-1c* (turquoise trace), *Napht-Chit-1d* (pink trace), *Napht-Chit-1e* (red trace).

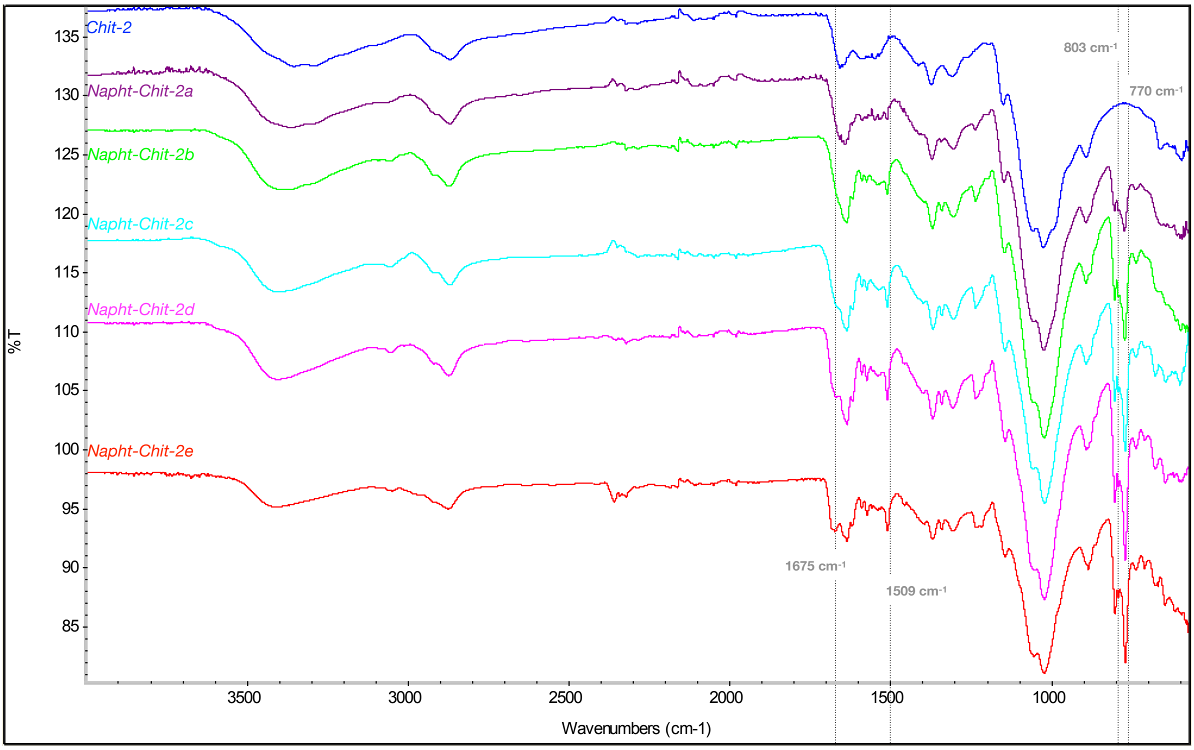

Figure S2. FTIR spectra of *Chit-2* and derivatives. From top to bottom: *Chit-2* (blue trace), *Napht-Chit-2a* (plum trace), *Napht-Chit-2b* (green trace), *Napht-Chit-2c* (turquoise trace), *Napht-Chit-2d* (pink trace), *Napht-Chit-2e* (red trace).

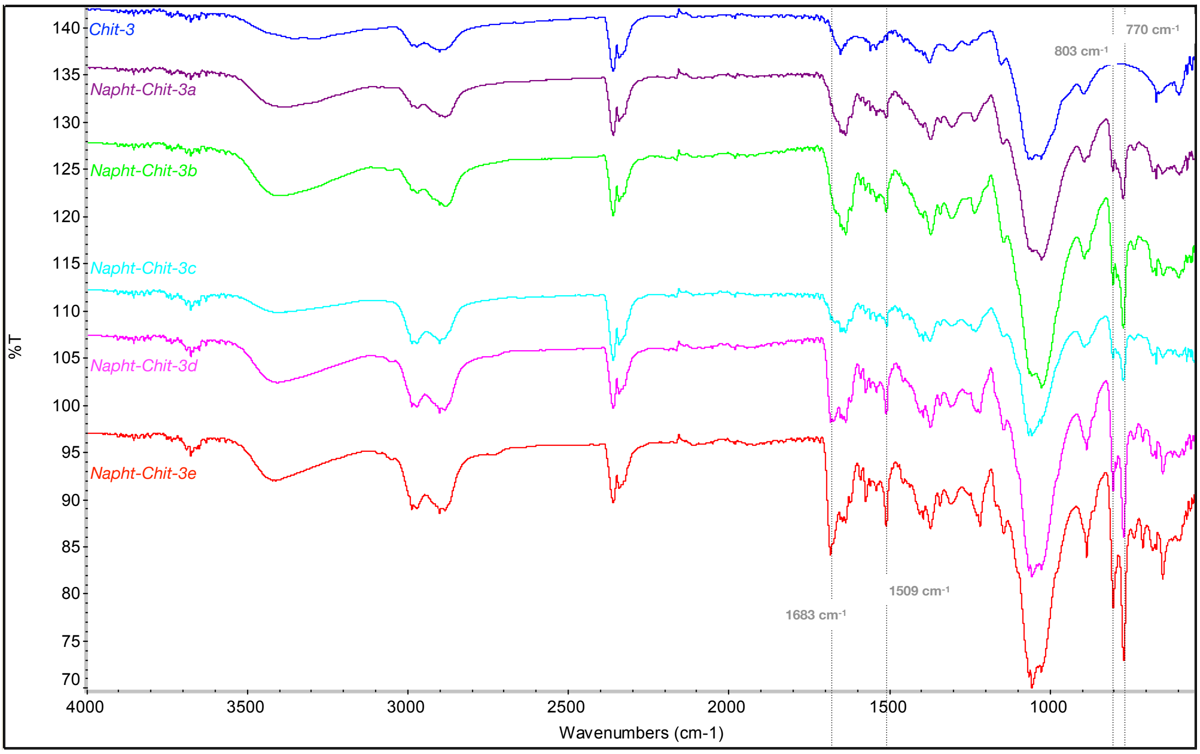

Figure S3. FTIR spectra of *Chit-3* and derivatives. From top to bottom: *Chit-3* (blue trace), *Napht-Chit-3a* (plum trace), *Napht-Chit-3b* (green trace), *Napht-Chit-3c* (turquoise trace), *Napht-Chit-3d* (pink trace), *Napht-Chit-3e* (red trace).

4. **Potentiometric titration of the chitosan resins.**

a. General principle

pH metric titration for unmodified and modified chitosan was made using an automatic titrator (848 Titrino plus, Metrohm). In brief, the chitosan resin is initially dissolved in HCl to protonate all the amine functionalities. Then, this solution is titrated with NaOH. The volume of sodium hydroxide used corresponds both to the volume necessary to neutralize the excess HCl acid used for dissolving the chitosan (V_1) and to neutralize the total acidity of the medium (V_2). The difference between V_2 and V_1 gives the quantity of NaOH necessary for the neutralization of the ammonium functions of the chitosan resins.

Figure S4 presents a titration curve for ChitoClear® HQG 400 (Table S3, Entry 19). Total titration time = 20 minutes. Another example of titration curve is presented in the manuscript (Figure 2).

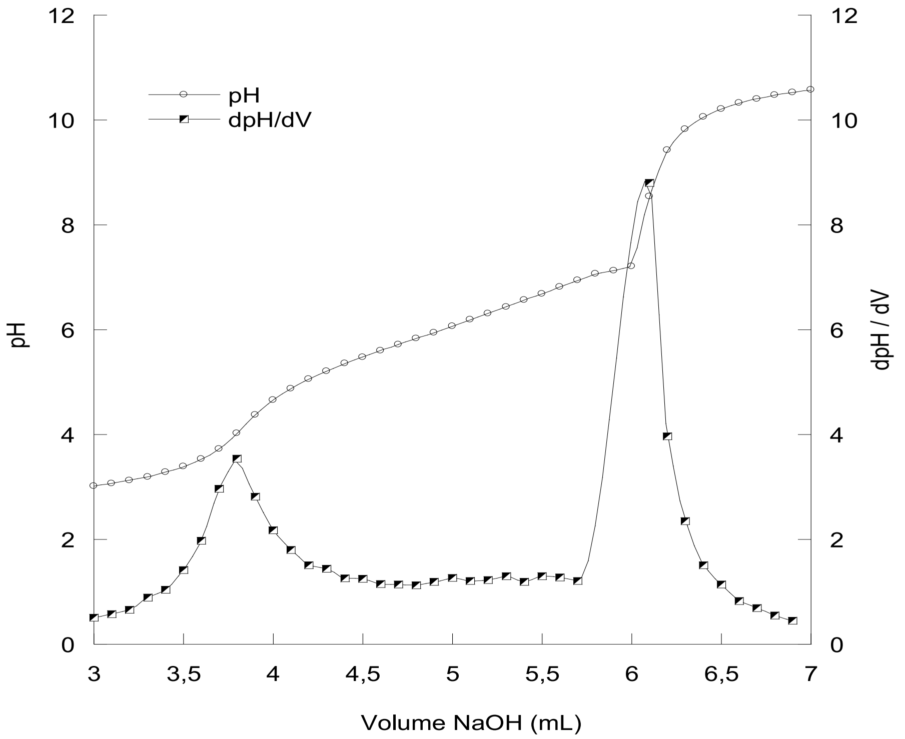

Figure S4. Potentiometric determination of *DDA* in starting material ChitoClear® HQG 400 (Table S3, Entry 19).

b. Titration results : complete raw values

Table S3 Titration results for all the chitosan resins involved in this study ([NaOH] = 0.10 mol/L)

Entry	Chitosan resin[I]	Mass used for titration (mg)	V_1 (mL)	V_2 (mL)
1	*Chit-1*	50.269	3.866	6.066
2		50.555	3.875	6.092
3		49.557	3.925	6.084
4	*Napht-Chit-1a*	50.050	4.288	6.164
5		50.579	4.260	6.180
6		50.200	4.250	6.160
7	*Napht-Chit-1b*	50.917	4.467	6.212
8		50.956	4.414	6.157
9		50.150	4.479	6.224
10	*Napht-Chit-1c*	50.144	4.547	6.151
11		50.665	4.591	6.235
12		50.285	4.589	6.171
13	*Napht-Chit-1d*	50.800	4.478	6.085
14		50.127	4.673	6.252
15		50.311	4.580	6.158
16	*Napht-Chit-1e*	50.864	4.668	6.205
17		50.192	4.661	6.158
18		50.503	4.687	6.224
19	*Chit-2*	49.656	3.844	6.073
20		50.18	3.878	6.175
21		49.774	3.819	6.086
22	*Napht-Chit-2a*	50.35	4.242	6.259
23		50.233	4.246	6.219
24		50.2	4.228	6.165
25	*Napht-Chit-2b*	50.269	4.382	6.140
26		50.072	4.16	5.840
27		49.75	4.397	6.177
28	*Napht-Chit-2c*	50.856	4.575	6.173
29		50.415	4.653	6.256
30		50.399	4.653	6.192
31	*Napht-Chit-2d*	50.495	4.742	6.159
32		50.976	4.763	6.171
33	*Napht-Chit-2e*	50.399	4.852	6.180
34		50.28	4.836	6.170

Table S3 (continued) Titration results for all the chitosan resins involved in this study ([NaOH] = 0.10 mol/L)

Entry	Chitosan resin[I]	Mass used for titration (mg)	V_1 (mL)	V_2 (mL)
35	*Chit-3*	50.243	4.049	6.051
36		50.162	4.062	6.062
37		50.076	4.046	6.053
38	*Napht-Chit-3a*	50.312	4.666	6.148
39		50.163	4.660	6.152
40		50.524	4.735	6.178
41	*Napht-Chit-3b*	50.282	4.769	6.114
42		50.449	4.813	6.154
43		50.623	4.803	6.157
44	*Napht-Chit-3c*	50.635	4.840	6.157
45		50.012	4.920	6.159
46		50.072	4.934	6.163
47	*Napht-Chit-3d*	50.431	3.4.956	6.155
48		50.077	4.960	6.159
49		50.788	4.962	6.150
50	*Napht-Chit-3e*	50.894	5.045	6.151
51		50.681	5.075	6.152
52		50.067	5.066	6.157

I. See Table 1 of the manuscript for details on those samples.

5. Determination of DDA from potentiometric titration: example

a. *Using Equation (1): example*

Let us use the first titration of *Napht-Chit-1a* as our example (Table S3, Entry 4). The experimental data of this titration are the following:

V_1 = 4.288 mL
V_2 = 6.164 mL.
ΔV = 1.876 mL.

m = 50.050 mg

[NaOH] = 0.10 mol/L

In the manuscript, we defined Equation (1) as:

$$DDA\ (\%) = 100\frac{(161.16\ \mathrm{g/mol} \cdot \Delta V \cdot [NaOH])}{m} \qquad \text{(S1)}$$

for which [NaOH] is the concentration (0.1 mol/L) of the sodium hydroxide solution used for titration, ΔV is the volume of HCl required to neutralize the ammonium functionalities (V_2-V_1, L), 161.16 (g/mol) is the molecular weight of the D-glucosamine monomer, and m is the mass (g) of the sample in the dry state before titration.

Applying Equation (S1) to our example gives:

$$DDA\ (\%) = 100\frac{(161.16\ \mathrm{g/mol} \cdot 1.876\ \cdot 10^{-3}\ \mathrm{L} \cdot 0.10\ \mathrm{mol/L})}{50.05 \cdot 10^{-3}\ \mathrm{g}}$$

$$DDA\ (\%) = 60.41$$

Titration was performed in triplicate for each chitosan resin.

The values were reported in the manuscript (Table 2) as means ± standard deviations.

For example, for *Napht-Chit-1a,* the three titrations gave the following *DDA* values.

60.41 (Table S3, Entry 4)
61.18 (Table S3, Entry 5)
61.32 (Table S3, Entry 6)

$$\mu = 60.97 \qquad \sigma = 0.49$$

Thus for this sample, the definitive value *DDA (%)* as calculated with Equation (1) is:

$$DDA\ (\%) = 61.0\ \pm 0.5$$

Complete detailed results can be found in Table S4.

b. Using Equation (2): example

Let us use again the first titration of *Napht-Chit-1a* as our example (Table S3, Entry 4). The experimental data of this titration are the following:

V_1 = 4.288 mL
V_2 = 6.164 mL.
ΔV = 1.876 mL.

m = 50.050 mg

[NaOH] = 0.10 mol/L

In the manuscript, we defined Equation (S2) as:

$$DDA\ (\%) = 100\frac{(203.19\ \text{g/mol} \cdot \Delta V \cdot [NaOH])}{m + (42.03\ \text{g/mol} \cdot \Delta V \cdot [NaOH])} \qquad \text{(S2)}$$

for which [NaOH] is the concentration (0.1 mol/L) of the sodium hydroxide solution used for titration, ΔV is the volume of HCl required to neutralize the ammonium functionalities (L), 203.19 (g/mol) is the molecular weight of the N-acetyl-glucosamine monomer, 42.033 (g/mol) is the difference between the molecular weight of N-acetyl-glucosamine monomer and that of D-glucosamine monomer, and m is the mass (g) of the sample in the dry state before titration.

Applying Equation (S2) to our example gives:

$$DDA\ (\%) = 100\frac{(203.19\ \text{g/mol} \cdot 1.876\ \cdot 10^{-3}\ \text{L} \cdot 0.10\ \text{mol/L})}{50.05 \cdot 10^{-3}\ \text{g} + (42.03\ \text{g/mol} \cdot 1.876\ \cdot 10^{-3}\ \text{L} \cdot 0.10\ \text{mol/L})}$$

$$DDA\ (\%) = 65.80$$

Titration was performed in triplicate for each chitosan resin.

The values were reported in the manuscript (Table 2) as means ± standard deviations.

For example, for *Napht-Chit-1a,* the three titrations gave the following *DDA* values.

65.80 (Table S3, Entry 4)
66.52 (Table S3, Entry 5)
66.65 (Table S3, Entry 6)

$$\mu = 66.32 \quad \sigma = 0.46$$

Thus for this sample, the definitive value *DDA* (%) as calculated with Equation (S1) is:

$$DDA\ (\%) = 66.3 \pm 0.5$$

Complete detailed results can be found in Table S4.

c. Calculating ΔDDA [Equation (2)-Equation (1)] : example

Let us use *Napht-Chit-1a again* as our example.

As detailed in the two precedent sections (5a and 5b), the *DDA* values (means of triplicate ± standard deviation) are as follows:

$$DDA_{Calulated\ with\ Equation\ (1)}\ (\%) = 61.0 \pm 0.5$$

$$DDA_{Calulated\ with\ Equation\ (2)}\ (\%) = 66.3 \pm 0.5$$

Consequently,

$$\Delta DDA\ [\text{Equation (2)} - \text{Equation (1)}](\%) = DDA_{Calulated\ with\ Equation\ (2)} - DDA_{Calulated\ with\ Equation\ (1)}\ (\%) = 66.3 - 61.0 = 5.3$$

The uncertainty associated with this value corresponds to the sum of the uncertainties on $DDA_{Calulated\ with\ Equation\ (1)}$ and $DDA_{Calulated\ with\ Equation\ (2)}$

Thus, for this sample, the definitive value for *ΔDDA* [Equation (S2) – Equation (S1)] is:

$$5 \pm 1$$

d. UsingEquation (17): example

Let us use again the first titration of *Napht-Chit-1a* as our example (Table S3, Entry 4). The experimental data of this titration are the following:

V_1 = 4.288 mL
V_2 = 6.164 mL.
ΔV = 1.876 mL.
m = 50.050 mg
[NaOH] = 0.10 mol/L

In the manuscript, we defined Equation (17) as:

$$DDA\ (\%) = 100\frac{(\Delta V \cdot [NaOH])\big((203.19\ \text{g/mol} \cdot DA) + (299.33\ \text{g/mol} \cdot DDA_{SM})\big)}{m_{chit} + (138.17\ \text{g/mol} \cdot \Delta V \cdot [NaOH])} \quad \text{(S17)}$$

for which ΔV is the difference between the two inflexion points (mL), [NaOH] is the concentration of the sodium hydroxide solution used for titration (mol /L), DDA_{SM} (%) is the degree of acetylation of the chitosan starting material (*Chit-1*, *Chit-2*, or *Chit-3*) used for modification as determined by Equation (S2) (see Table 2), DA is the degree of acetylation (%) of the resin (DA = 100% - DDA_{SM}), 203.19 g/mol is the molecular weight of the N-acetylglucosamine monomer, 299.33 g/mol is the molecular weight of the modified monomer (*N-Napht-glucosamine*), m_{Chit} is the mass of the chitosan sample used in the titration (g), and 138.17 g/mol is the difference between the molar mass of the modified monomer and the D-glucosamine monomer (299.33 g/mol – 161.16 g/mol).

For *Napht-Chit-1a*, we also know that:

DDA_{SM} = 75.1% ± 0.2% [*DDA* for *Chit-1* calculated with Equation (2)]
DA = 24.9% ± 0.2% [100% – 75.1% = 24.9%]

Applying Equation (S17) to our example gives:

$$DDA\ (\%) = 100\frac{(1.876\ \cdot 10^{-3}\ \mathrm{L} \cdot 0.1\ \mathrm{mol/L})\big((203.19\ \mathrm{g/mol} \cdot 24.9\%) + (299.33\ \mathrm{g/mol} \cdot 75.1\%)\big)}{50.050\ \cdot 10^{-3}\mathrm{g} + (138.17\ \mathrm{g/mol} \cdot 1.876\ \cdot 10^{-3}\ \mathrm{L} \cdot 0.1\ \mathrm{mol/L})}$$

$$DDA\ (\%) = 68.00$$

Uncertainties in the *DDA* values obtained from Equation (S17) were calculated considering the uncer-tainties on DDA_{SM} and *DA* using the upper-lower bound method of uncertainty propagation.

To determine the minimum and maximum values, we also kept in mind that, by definition:

$DA + DDA_{SM} = 100\%$

For our example above, this gives us:

$$DDA_{Max}\ (\%) = 100\frac{(1.876\ \cdot 10^{-3}\ \mathrm{L} \cdot 0.1\ \mathrm{mo}l\mathrm{/L})\big((203.19\ \mathrm{g/mol} \cdot (24.9\% - 0.2\%) + (299.33\ \mathrm{g/mol} \cdot 75.1\% + 0.2\%))\big)}{50.050\ \cdot 10^{-3}\mathrm{g} + (138.17\ \mathrm{g/mol} \cdot 1.876\ \cdot 10^{-3}\ \mathrm{L} \cdot 0.1\ \mathrm{mol/L})}$$

$$DDA_{Max}\ (\%) = 100\frac{(1.876\ \cdot 10^{-3}\ \mathrm{L} \cdot 0.1\ \mathrm{mol/L})\big((203.19\ \mathrm{g/mol} \cdot 24.7\%) + (299.33\ \mathrm{g/mol} \cdot 75.3\%)\big)}{50.050\ \cdot 10^{-3}\mathrm{g} + (138.17\ \mathrm{g/mol} \cdot 1.876\ \cdot 10^{-3}\ \mathrm{L} \cdot 0.1\ \mathrm{mol/L})}$$

$$DDA_{Max}\ (\%) = = 68.05$$

$$DDA_{Min}\ (\%) = 100\frac{(1.876\ \cdot 10^{-3}\ L \cdot 0.1\ \mathrm{mol/L})\big((203.19\ \mathrm{g/mol} \cdot (24.9\% + 0.2\%) + (299.33\ \mathrm{g/mol} \cdot 75.1\% - 0.2\%))\big)}{50.050\ \cdot 10^{-3}\mathrm{g} + (138.17\ \mathrm{g/mol} \cdot 1.876\ \cdot 10^{-3}\ \mathrm{L} \cdot 0.1\ \mathrm{mol/L})}$$

$$DDA_{Max}\ (\%) = 100\frac{(1.876\ \cdot 10^{-3}\ \mathrm{L} \cdot 0.1\ mol/L)\big((203.19\ \mathrm{g/mol} \cdot 25.1\%) + (299.33\ \mathrm{g/mol} \cdot 74.9\%)\big)}{50.050\ \cdot 10^{-3}g + (138.17\ \mathrm{g/mol} \cdot 1.876\ \cdot 10^{-3}\ \mathrm{L} \cdot 0.1\ \mathrm{mol/L})}$$

$$DDA_{Max}\ (\%) = = 67.95$$

Thus for this sample, the definitive value for *DDA* calculated with Equation (S17) is:

$$DDA\ (\%) = 68.00\ \pm 0.05$$

To calculate the *DDA* for the whole triplicate, we calculated the mean. Uncertainties were obtained by considering the maximum and minimum values for each term and calculating the standard deviation.

For example, for *Napht-Chit-1a* (Table S4, Entry 4,5 and 6)

DDA (%) = 68.00 ± 0.05 (Table S4, Entry 4)
DDA (%) = 68.57 ± 0.05 (Table S4, Entry 5)
DDA (%) = 68.68 ± 0.05 (Table S4, Entry 6)

$$\mu = \frac{68.00 + 68.57 + 68.68}{3} = 68.42$$

$$\sigma = \sqrt{\frac{(\mu - 68.00)^2 + (\mu - 67.95)^2 + (\mu - 68.05)^2 + (\mu - 68.57)^2 + (\mu - 68.52)^2 + (\mu - 68.62)^2 + (\mu - 68.68)^2 + (\mu - 68.63)^2 + (\mu - 68.73)^2}{9}}$$

$$= 0.3$$

Thus, for *Napht-Chit-1a* the final value of *DDA* calculated with Equation (S17) and reported in Table 4 of the manuscript is :

DDA (%) = 68.4 ± 0.3

e. Calculating ΔDDA [Equation (S17)-Equation (S2)] : example

Let us use *Napht-Chit-1a again* as our example.

As detailed in the preceding sections (5b and 5d), the *DDA* values (means of triplicate ± standard deviation) are as follows:

$$DDA_{Calulated\ with\ Equation\ \text{(S2)}}\ (\%) = 66.3 \pm 0.5$$

$$DDA_{Calulated\ with\ Equation\ \text{(S17)}}\ (\%) = 68.4 \pm 0.3$$

Consequently,

$$\Delta DDA\ \text{[Equation (S17)} - \text{Equation (S2)](\%)} = DDA_{Calulated\ with\ Equation\ \text{(17)}} - DDA_{Calulated\ with\ Equation\ \text{(2)}}\ (\%) = 68.4 - 66.3 = 2.1$$

The uncertainty associated with this value corresponds to the sum of the uncertainties on $DDA_{Calulated\ with\ Equation\ \text{(S17)}}$ and $DDA_{Calulated\ with\ Equation\ \text{(S2)}}$

Thus, for this sample, the definitive value for *ΔDDA* [Equation (S17)-Equation (S2)] as indicated in Table 4 of the manuscript is:

$$2.1 \pm 0.8$$

f. Complete results

Table S4 Determination of *DDA* values for all the chitosan resins involved in this study as determined by potentiometric titration after calculation with Equation (S1), Equation (S2) and Equation (S17): complete detailed results.

Entry	Chitosan resin	DDA_{SM} (%)	*DA* (%)	m (mg)	V_1 (mL)	V_2 (mL)	*DDA* (%) calculated with Equation (S1)		*DDA* (%) calculated with Equation (S2)		*DDA* (%) calculated with Equation (S17)	
							triplicate	mean	triplicate	mean	triplicate	mean
1	*Chit-1*	n/a	n/a	50.269	3.866	6.066	70.53	70.5 ± 0.2	75.11	75.1 ± 0.2	n/a	n/a
2				50.555	3.875	6.092	70.67		75.24		n/a	
3				49.557	3.925	6.084	70.21		74.82		n/a	
4	*Napht-Chit-1a*	75.1 ± 0.2	75.1 ± 0.2	50.050	4.288	6.164	60.41	61.0 ± 0.5	65.80	66.3 ± 0.5	68.00 ± 0.05	68.4 ± 0.3
5				50.579	4.260	6.180	61.18		66.52		68.57 ± 0.05	
6				50.200	4.250	6.160	61.32		66.65		68.68 ± 0.05	
7	*Napht-Chit-1b*	75.1 ± 0.2	75.1 ± 0.2	50.917	4.467	6.212	55.23	55.5 ± 0.5	60.87	61.1 ± 0.5	64.05 ± 0.04	64.2 ± 0.3
8				50.956	4.414	6.157	55.13		60.77		63.97 ± 0.04	
9				50.150	4.479	6.224	56.08		61.68		64.71 ± 0.04	
10	*Napht-Chit-1c*	75.1 ± 0.2	75.1 ± 0.2	50.144	4.547	6.151	51.55	51.5 ± 0.8	57.29	57.3 ± 0.8	61.09 ± 0.04	61.1 ± 0.6
11				50.665	4.591	6.235	52.29		58.02		61.70 ± 0.04	
12				50.285	4.589	6.171	50.70		56.46		60.39 ± 0.04	
13	*Napht-Chit-1d*	75.1 ± 0.2	75.1 ± 0.2	50.800	4.478	6.085	50.98	50.8 ± 0.2	56.73	56.5 ± 0.2	60.62 ± 0.04	60.4 ± 0.2
14				50.127	4.673	6.252	50.77		56.52		60.44 ± 0.04	
15				50.311	4.580	6.158	50.55		56.31		60.26 ± 0.04	
16	*Napht-Chit-1e*	75.1 ± 0.2	75.1 ± 0.2	50.864	4.668	6.205	48.70	48.6 ± 0.5	54.48	54.4 ± 0.5	58.71 ± 0.04	58.6 ± 0.4
17				50.192	4.661	6.158	48.07		53.85		58.17 ± 0.04	
18				50.503	4.687	6.224	49.05		54.83		59.00 ± 0.04	

Table S4 (continued) Determination of *DDA* values for all the chitosan resins involved in this study as determined by potentiometric titration after with Equation calculations (S1), Equation (S2) and Equation (S17): complete detailed results.

Entry	Chitosan resin	DDA_{SM} (%)	*DA* (%)	m (g)	V_1 (mL)	V_2 (mL)	*DDA* (%) calculated with Equation (S1)		*DDA* (%) calculated with Equation (S2)		*DDA* (%) calculated with Equation (S17)	
							triplicate	mean	triplicate	mean	triplicate	mean
19				49.656	3.844	6.073	72.34		76.73		n/a	n/a
20	*Chit-2*	n/a	n/a	50.18	3.878	6.175	73.77	73.2 ± 0.7	79.00	77.5 ± 0.7	n/a	
21				49.774	3.819	6.086	73.40		77.67		n/a	
22				50.35	4.242	6.259	64.56		69.67		71.6 ± 0.2	
23	*Napht-Chit-2a*	77.5 ± 0.7	22.5 ± 0.7	50.233	4.246	6.219	63.30	63 ± 1	68.50	68 ± 1	70.7 ± 0.2	70.7 ± 0.8
24				50.2	4.228	6.165	62.02		67.31		69.8 ± 0.2	
25				50.269	4.382	6.140	56.36		61.95		65.5 ± 0.2	
26	*Napht-Chit-2b*	77.5 ± 0.7	22.5 ± 0.7	50.072	4.16	5.840	53.94	56 ± 2	59.62	62 ± 2	63.6 ± 0.1	65 ± 1
27				49.75	4.397	6.177	57.66		63.20		66.5 ± 0.2	
28				50.856	4.575	6.173	50.64		56.40		60.8 ± 0.1	
29	*Napht-Chit-2c*	77.5 ± 0.7	22.5 ± 0.7	50.415	4.653	6.256	51.24	50± 1	56.99	56 ± 1	61.3 ± 0.1	60.6 ± 0.8
30				50.399	4.653	6.192	49.21		54.99		59.6 ± 0.1	
31	*Napht-Chit-2d*	77.5 ± 0.7	22.5 ± 0.7	50.495	4.742	6.159	45.22	44.9 ± 0.5	51.00	50.6 ± 0.5	56.1 ± 0.1	55.8 ± 0.4
32				50.976	4.763	6.171	44.51		50.28		55.5 ± 0.1	
33	*Napht-Chit-2e*	77.5 ± 0.7	22.5 ± 0.7	50.399	4.852	6.180	42.46	42.6 ± 0.2	48.20	48.4 ± 0.2	53.6 ± 0.1	53.8 ± 0.2
34				50.28	4.836	6.170	42.76		48.50		53.9 ± 0.1	

Table S4 (continued) Determination of *DDA* values for all the chitosan resins involved in this study, as determined by potentiometric titration after calculations with Equation (S1), Equation (S2) and Equation (S17): complete detailed results.

Entry	Chitosan resin	DDA_{SM} (%)	*DA* (%)	m (g)	V_1 (mL)	V_2 (mL)	*DDA* (%) calculated with Equation (S1)		*DDA* (%) calculated with Equation (S2)		*DDA* (%) calculated with Equation (S17)	
							triplicate	mean	triplicate	mean	triplicate	mean
35	*Chit-3*	n/a	n/a	50.243	4.049	6.051	64.22	64.4± 0.2	69.35	69.5 ± 0.2	n/a	n/a
36				50.162	4.062	6.062	64.26		69.39		n/a	
37				50.076	4.046	6.053	64.59		69.7		n/a	
38	*Napht-Chit-3a*	69.5 ± 0.2	30.5 ± 0.2	50.312	4.666	6.148	47.47	47 ± 1	53.26	53 ± 1	56.53 ± 0.04	56.2 ± 0.7
39				50.163	4.660	6.152	47.93		53.72		56.92 ± 0.04	
40				50.524	4.735	6.178	46.03		51.81		55.29 ± 0.04	
41	*Napht-Chit-3b*	69.5 ± 0.2	30.5 ± 0.2	50.282	4.769	6.114	43.11	43.0 ± 0.2	48.86	48.8 ± 0.2	52.73 ± 0.04	52.6 ± 0.1
42				50.449	4.813	6.154	42.84		48.58		52.49 ± 0.04	
43				50.623	4.803	6.157	43.1		48.85		52.73 ± 0.04	
44	*Napht-Chit-3c*	69.5 ± 0.2	30.5 ± 0.2	50.635	4.840	6.157	41.92	40 ± 1	47.64	46 ± 1	51.66 ± 0.04	50 ± 1
45				50.012	4.920	6.159	39.93		45.59		49.83 ± 0.03	
46				50.072	4.934	6.163	39.56		45.21		49.49 ± 0.03	
47	*Napht-Chit-3d*	69.5 ± 0.2	30.5 ± 0.2	50.431	3.4.956	6.155	38.32	38.2 ± 0.5	43.92	43.8 ± 0.5	48.32 ± 0.03	48.2 ± 0.4
48				50.077	4.960	6.159	38.59		44.2		48.58 ± 0.03	
49				50.788	4.962	6.150	37.7		43.27		47.73 ± 0.03	
50	*Napht-Chit-3e*	69.5 ± 0.2	30.5 ± 0.2	50.894	5.045	6.151	35.02	34.8 ± 0.5	40.46	40.2 ± 0.5	45.13 ± 0.03	44.9 ± 0.4
51				50.681	5.075	6.152	34.25		39.64		44.35 ± 0.03	
52				50.067	5.066	6.157	35.12		40.56		45.22 ± 0.03	
